# Tutorial on phantoms for photoacoustic imaging applications

**DOI:** 10.1117/1.JBO.29.8.080801

**Published:** 2024-08-14

**Authors:** Lina Hacker, James Joseph, Ledia Lilaj, Srirang Manohar, Aoife M. Ivory, Ran Tao, Sarah E. Bohndiek, Members of IPASC

**Affiliations:** a University of Oxford, Department of Oncology, Oxford, United Kingdom; b University of Dundee, School of Science and Engineering, United Kingdom; c University of Dundee, Centre for Medical Engineering and Technology, Dundee, United Kingdom; d iThera Medical GmbH, Munich, Germany; e University of Twente, Tech Med Centre, Multi-Modality Medical Imaging, Enschede, The Netherlands; f St. Vincent’s Private Hospital, Department of Medical Physics, Dublin, Ireland; g University of Cambridge, Department of Physics, Cambridge, United Kingdom; h University of Cambridge, Cancer Research UK Cambridge Institute, Cambridge, United Kingdom

**Keywords:** phantom, standardization, photoacoustic imaging, calibration

## Abstract

**Significance:**

Photoacoustic imaging (PAI) is an emerging technology that holds high promise in a wide range of clinical applications, but standardized methods for system testing are lacking, impeding objective device performance evaluation, calibration, and inter-device comparisons. To address this shortfall, this tutorial offers readers structured guidance in developing tissue-mimicking phantoms for photoacoustic applications with potential extensions to certain acoustic and optical imaging applications.

**Aim:**

The tutorial review aims to summarize recommendations on phantom development for PAI applications to harmonize efforts in standardization and system calibration in the field.

**Approach:**

The International Photoacoustic Standardization Consortium has conducted a consensus exercise to define recommendations for the development of tissue-mimicking phantoms in PAI.

**Results:**

Recommendations on phantom development are summarized in seven defined steps, expanding from (1) general understanding of the imaging modality, definition of (2) relevant terminology and parameters and (3) phantom purposes, recommendation of (4) basic material properties, (5) material characterization methods, and (6) phantom design to (7) reproducibility efforts.

**Conclusions:**

The tutorial offers a comprehensive framework for the development of tissue-mimicking phantoms in PAI to streamline efforts in system testing and push forward the advancement and translation of the technology.

## 1 Introduction

Central to the process of successfully translating a novel medical imaging modality lies accurate characterization of device performance and standardization of measurements. For this purpose, test objects with well-defined properties are used to relate a measured imaging signal to the underlying ground-truth property. A subset of these test objects is referred to as tissue-mimicking phantoms, which are artificial objects specifically designed to mimic certain properties of real-world tissues, objects or materials for calibration, testing, and validation purposes. Phantoms are essential to establish consensus performance test methods and support future standards development in biomedical imaging, enabling quality assurance and control as well as performance comparison of different imaging equipment during development and marketing.

But what qualities define a ‘good’ phantom? Which design parameters are essential? The International Photoacoustic Standardisation Consortium (IPASC)^1^ has compiled consensus recommendations on characteristics for an ideal (physical) phantom for the emerging imaging modality of photoacoustic imaging (PAI). This tutorial summarizes these recommendations in a seven-step framework, guiding through the process of understanding and defining relevant terminology and parameters (steps 1 and 2), defining the purpose of a phantom (step 3), identifying relevant material properties and characterization methods (steps 4 and 5), creating a suitable phantom design (step 6), and ensuring reproducibility (step 7). A glossary of relevant terms related to standardization that are used in this document can be found in Table 1.

**Table 1:** Glossary. Definitions of key terminology.

Term	Definition
Accuracy (bias)	Accuracy refers to the closeness of measurements to the true value. Bias is the systematic deviation of measurements from the true value.
Benchmarking	Benchmarking is the process of comparing the performance of a system, method, or product against recognized standards or best practices.
Calibration	Calibration involves adjusting or standardizing measurement devices or instruments to ensure their accuracy and reliability. In biomedical imaging, calibration ensures that the imaging system provides measurements that are traceable to known standards.
Precision	Precision relates to the degree of consistency in repeated measurements. It quantifies the variability among multiple measurements of the same quantity.
Quality	The degree to which a product, process, or system meets defined standards and satisfies customer requirements.
Quality assurance/quality control (QA/QC)	QA involves systematic activities and processes that ensure quality standards are met throughout a project. QC involves specific measures taken to monitor and control the quality of processes and outputs.
Reference	A known standard or measurement used for comparison or calibration. In biomedical imaging, reference images or measurements are used to validate and calibrate imaging systems, ensuring that the results are accurate and consistent.
Repeatability	A measure of the extent to which a test conducted multiple times on the same subject, in the same lab, using the same equipment, by the same operator, over a short period of time, should give the same result.^2^
Reproducibility	A measure of the extent to which a test conducted multiple times in different labs, using different equipment, by different operators, or over different periods of time, should give comparable results.^2^
Validation	The process of assessing the accuracy, reliability, and suitability of a method or system for its intended use.
Verification	The process of evaluating whether a specific system, method, or process meets predetermined specifications and fulfils its intended purpose.

## 2 Main

### 2.1 Step 1: Understanding the Concept of the Imaging Modality

**Fig. 1:**
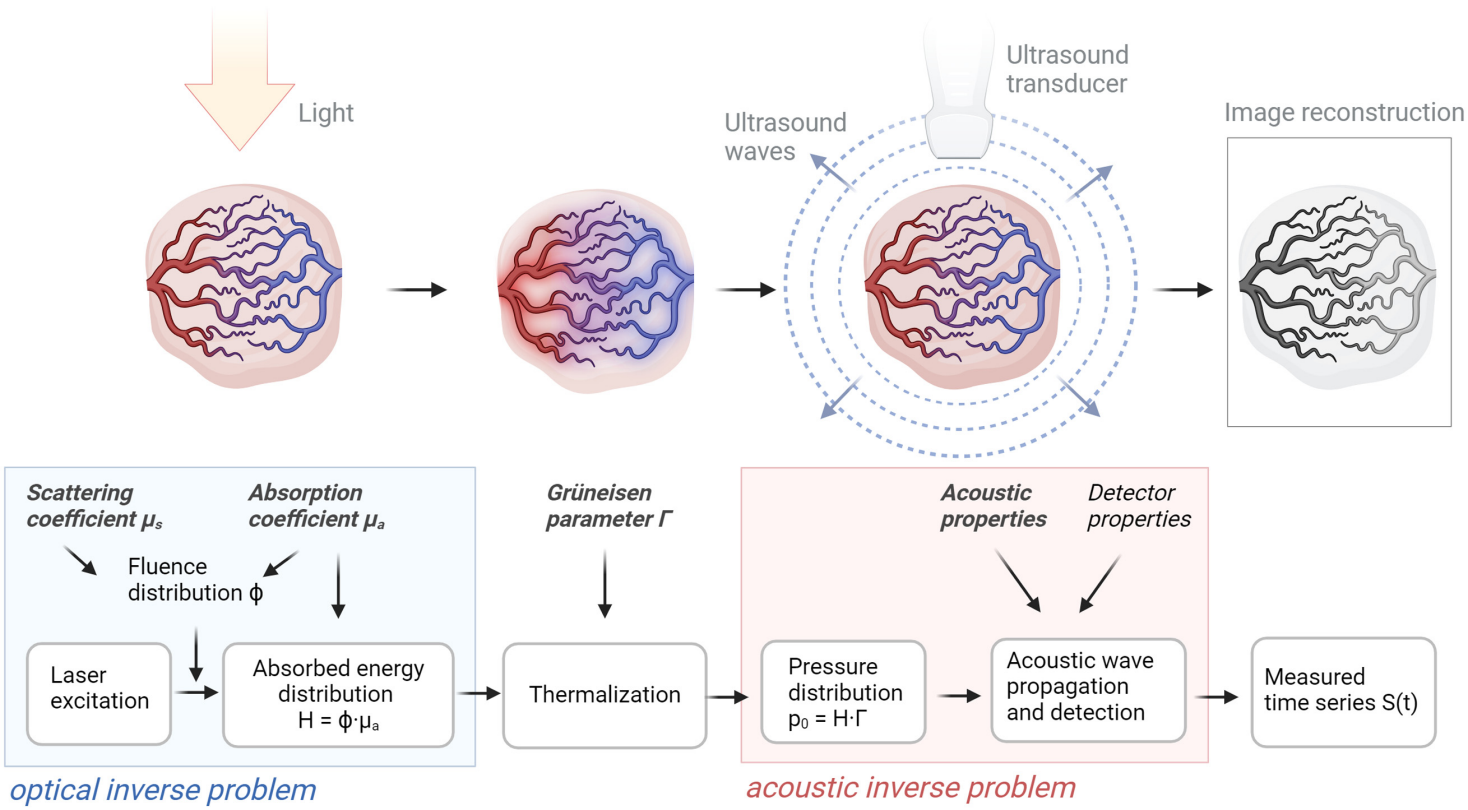
Photoacoustic signal generation. A pulsed light source illuminates the object to be imaged (e.g., tumor tissue). As the light propagates through the tissue, it is scattered and absorbed by spatially varying endogenous or exogenous chromophores. The absorption and scattering coefficients, $\mu_{a}$ and $\mu_{s}$, determine the fluence distribution Φ and consequently, the absorbed energy distribution H. The absorbed energy generates a pressure distribution $p_{0}$. The combined photoacoustic efficiency of conversion from heat into pressure is represented by the Grüneisen parameter Γ. Due to the elastic nature of the tissue, the generated pressure propagates as an acoustic wave through the tissue and is detected by ultrasound sensors. This process is affected by the acoustic properties of the tissue and the sensor response. Finally, image reconstruction is performed to visualize the recorded data. Created with BioRender.

The first step in developing robust measures to test medical imaging systems is a comprehensive understanding of the underlying principles driving the imaging modality of interest and its source of contrast. For PAI, the signal is generated through the photoacoustic effect, which refers to the generation of ultrasound waves from the absorption of electromagnetic energy^3^ (Fig. 1): short light pulses, typically in the nanosecond range, illuminate a sample and the absorption of energy by the imaged object results in heat generation; the rise in temperature leads to an increase in pressure, which generates broadband ultrasound waves that propagate through the tissue and are detected by ultrasound transducers. The amplitude of the recorded pressure wave provides information about the local absorption of optical energy within the object. The time interval between the illumination pulse and the arrival of the ultrasound wave at the detector can be used to calculate the distance between the detector and the absorbers.

**Fig. 2:**
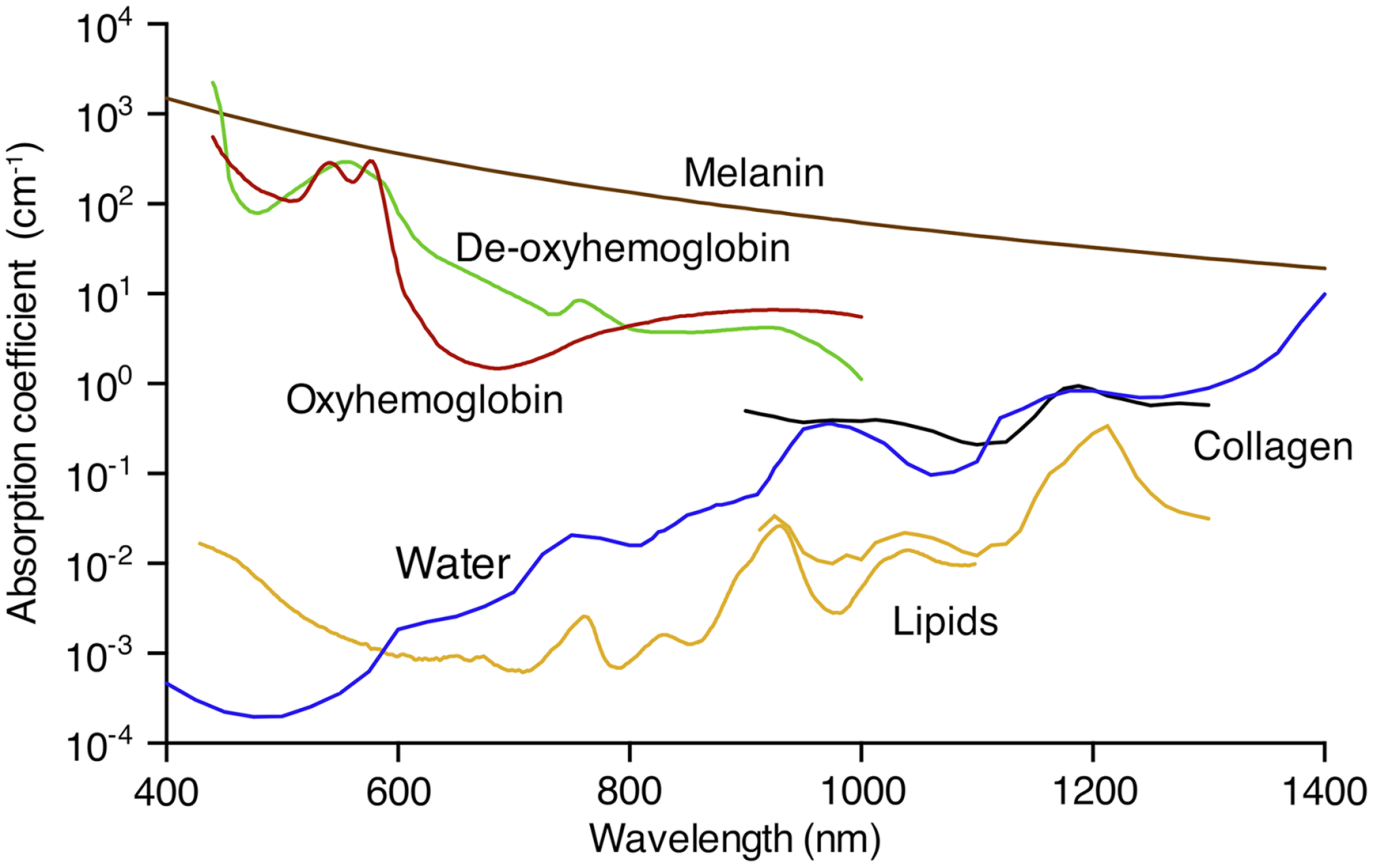
Absorption spectra of the main endogenous chromophores. Absorption spectra are displayed for melanin (brown); oxy- (red) and deoxy-hemoglobin (green; both $150\;{\mathrm{gl}}^{-1}$); water (blue; 80% by volume in tissue); lipids (yellow; 20% by volume in tissue); and collagen (black) in the wavelength range of 400 to 1400 nm. Data from Refs. 7 and 8.

For illumination in tissues, light in the near-infrared (NIR, 650 to 1350 nm) window is often chosen to optimize penetration depth.^4^ Applying pulses at multiple wavelengths enables multispectral photoacoustic systems to distinguish between endogenous chromophores or exogenous contrast agents, such as nanoparticles or organic dyes.^5^^,^^6^ The primary endogenous absorbers in tissue are oxy- and deoxy-hemoglobin (${\mathrm{HbO}}_{2}$ and RHb), lipids, melanin, collagen, and water (Fig. 2). The optical absorption coefficients vary with wavelength, and thus the relative concentration of each chromophore can be extracted through spectroscopic inversion. Functional parameters such as haemoglobin oxygen saturation (${\mathrm{sO}}_{2}$) can be calculated from the absorption difference between ${\mathrm{HbO}}_{2}$ and RHb (${\mathrm{sO}}_{2}={\mathrm{HbO}}_{2}/({\mathrm{HbO}}_{2}+\mathrm{RHb})$).^9^^,^^10^ Using only endogenous contrast, PAI therefore has the potential to non-invasively visualize simultaneously various important tissue parameters within a single imaging session. Current state-of-the-art technology can achieve two-dimensional imaging in almost real-time, and three-dimensional (3D) images in time scales of seconds to minutes.^5^^,^^11^^,^^12^ Importantly, the modality is also able to image across scales, from whole tumor volumes (macroscopic, cm depth) over to vascular networks (mesoscopic, mm depth) down to individual cells (microscopic, μm depth)^11^ simply by scaling the system configurations. Providing 3D multi-parametric information with high temporal resolution, PAI has found a wide range of applications in both pre-clinical and clinical environments.^13^

### 2.2 Step 2: Defining Relevant Parameters and Their Magnitude

Following a thorough comprehension of the imaging modality, the parameters that play a role in the signal generation process need to be precisely defined and their magnitude well understood to design and develop suitable phantoms for the modality (Table 2). For PAI, optical, acoustic, and (thermo)mechanical properties are paramount. To characterize light propagation, knowledge of the probability of a scattering or absorption event per unit path length is essential. These parameters are captured by the linear absorption coefficient $\mu_{a}(\lambda )$ and linear scattering coefficient $\mu_{s}(\lambda )$. For scattering events where the source-to-measurement distance is much larger than $1{/\mu }_{s}(\lambda )$, the reduced scattering coefficient ${\mu_{s}}^{\prime }(\lambda )$ is used to describe scattering behavior. The reduced scattering coefficient is defined by ${\mu_{s}}^{\prime }(\lambda )=(1-g)\mu_{s}(\lambda )$ where g is the anisotropy factor, a parameter that indicates how scattering intensity varies with angle. For PAI phantom applications, the anisotropy factor plays a role in microscopic or mesoscopic applications or interactions near a light source. In addition, the refractive index n is relevant for light reflection and refraction at interfaces. Scattering and absorption effects are wavelength-dependent and determined by the constituents of the medium.

**Table 2:** Definition of relevant material properties parameters in optical and acoustic imaging applications. The soft tissue values are just representative values. The properties may vary with the experimental conditions (e.g., temperature). See literature for values that pertain to specific samples and controls.

	Parameter	Symbol	Unit	Definition	Range in soft tissue	Ref.
Optical	Optical absorption coefficient	$\mu_{a}$	${\mathrm{cm}}^{-1}$	Probability of photon absorption per unit length traveled by the photon	0.1 to 0.5	^8^ ^,^ ^14^
	Refractive index	n	—	Ratio of the velocity of light in a vacuum to the velocity of light in a medium	1.33 to 1.51	^8^ ^,^ ^14^
	Anisotropy factor	g	—	Average of the cosine of the scattering angle, which represents the effects of directionally dependent scattering	0.7 to 0.9	^8^ ^,^ ^14^
	Reduced scattering coefficient	${\mu_{s}}^{\prime }$	${\mathrm{cm}}^{-1}$	Probability of photon scattering per unit length travelled by the photon	10 to 20	^8^ ^,^ ^14^
Acoustic	Acoustic attenuation coefficient	α	$\mathrm{dB}\cdot {\mathrm{cm}}^{-1}\cdot {\mathrm{MHz}}^{-y}$	Extent of reduction of an acoustic wave when propagating within a medium	0.1 to 1.6	^15^
	Speed of sound	v	$m\cdot s^{-1}$	Speed of acoustic wave propagation within a medium	1450 to 1730	^16^
	Backscattering coefficient	$\mu_{bs}$	$m^{-1}\;{\mathrm{sr}}^{-1}$	Differential scattering cross section per unit volume at a scattering angle of 180 deg	$3.5\times 10^{-4}$ to $9\times 10^{-4}$	^17^
Mechanical	Density	ρ	${\mathrm{kg}\cdot m}^{-3}$	Measure of mass per unit volume	0.95 to 1.15	^16^
	Young’s modulus	E	$N\cdot m^{-2}$	Ratio of stress applied to a material to the strain that results from the stress	$1\times 10^{2}$ to $1\times 10^{6}$	^16^
Thermoelastic	Grüneisen parameter	Γ	—	Measure of thermoelastic efficiency	0.25 to 0.9	^18^ ^,^ ^19^

When light is converted into heat and subsequently into a pressure wave, the efficiency of the conversion is represented by the Grüneisen parameter Γ. In order to assess how the generated pressure wave propagates within a medium, a well-characterized broadband frequency description of the material-specific speed of sound c and acoustic attenuation coefficient α is essential. Other acoustic parameters, such as the acoustic backscattering coefficient $\mu_{bs}$ or the ultrasound non-linearity parameter (B/A), are less reported in PAI, as the key interest lies in determining the amplitude of the acoustic losses than analyzing the fate of the encountered losses. The propagation of acoustic waves is determined by the mechanical properties of the medium, specifically by its density and elastic properties (quantified by the Young’s modulus/shear modulus).

Average values for the optical,^15^^,^^20^ acoustic,^16^^,^^21^ and thermoelastic^14^ properties of soft tissues are summarized in Table 3. It is worth noting that methods for measuring these parameters across different tissue types can often require complex equipment that must be independently calibrated, which is particularly challenging for some parameters, e.g., the acoustic backscattering coefficient or the Grüneisen parameter.

**Table 3:** Overview of representative acoustic and optical properties found in soft tissues. Optical properties cover a spectrum from 600 to 900 nm. For values outside this wavelength range, please refer to the literature.^20^

Tissue type	Acoustic properties		Optical properties		Ref.
	v ($m\cdot s^{-1}$)	α ($\mathrm{dB}\cdot {\mathrm{cm}}^{-1}$) at f (MHz)	$\mu_{a}$ (${\mathrm{cm}}^{-1}$)	${\mu_{s}}^{\prime }$ (${\mathrm{cm}}^{-1}$)	
Soft tissue	1450 to 1575	0.5 to 30 at 1 to 10 MHz	0.1 to 0.5	10 to 20	22, 23
Breast fat	1430 to 1480	1 to 18 at 1 to 10 MHz	0.05 to 0.4	3 to 8	22, 23
Breast parenchyma	1460 to 1520	2 to 25 at 1 to 10 MHz	0.1 to 0.3	5 to 15	22
Blood	1560 to 1570	0.1 to $0.2\;\mathrm{dB}\cdot {\mathrm{cm}}^{-1}\cdot {\mathrm{MHz}}^{-1}$	2.0 to 10.0	10 to 15	20, 24, 25
Brain	1550	0.6 at 1 MHz	0.2 to 9	8 to 90	21, 25, 26
Liver	1510 to 1590	0.5 to $0.9\;\mathrm{dB}\cdot {\mathrm{cm}}^{-1}\cdot {\mathrm{MHz}}^{-1}$	1.15 to 1.56	22 to 30	20, 24, 27
Prostate	1614	$1.86\;\mathrm{dB}\cdot {\mathrm{cm}}^{-1}\cdot {\mathrm{MHz}}^{-1}$	0.05 to 0.72	1 to 40	20, 25
Skin	∼1600	2 to $4\;\mathrm{dB}\cdot {\mathrm{cm}}^{-1}\cdot {\mathrm{MHz}}^{-1}$	0.05 to 1.11	2 to 21	20, 25, 27
Muscle	1540 to 1580	1.3 to 3.3 at 1 MHz	0.05 to 0.17	6 to 10	20, 24
Tendon	1670	$4.7\;\mathrm{dB}\cdot {\mathrm{cm}}^{-1}\cdot {\mathrm{MHz}}^{-1}$	^a^	—	25
Water	1480	0.0022 at 1 MHz	0.006 to 0.07	0.003	25, 28

a No specific reference found.

### 2.3 Step 3: Defining the Purpose of the Phantom

For the creation of high-performing phantoms, it is imperative to thoroughly comprehend their intended objectives and functions. Phantoms fulfil various tasks along the full translational pipeline of an imaging modality^29^^,^^30^ including:

• Quantitatively and objectively assessing the performance of technologies across multiple stages of their development;
• Technically validating device performance in controlled test-bed environments (routine quality control), e.g., assessing drift in accuracy and precision of the imaging device (over time, device-to-device, and site-to-site variations);
• Facilitating system design optimization (hardware or software); assessing impact of system upgrades;
• Comparison between different diagnostic modalities;
• User training;
• Marketing;
• Technical demonstrations;
• Testing to support regulatory evaluation; benchmarking.

Phantoms are often designed to meet one or several of these purposes, which dictates their final design and intrinsic properties.

### 2.4 Step 4: Defining Desired Properties of a Phantom Material

The ideal properties of a phantom are specified by its final application. For example, phantoms that are targeted toward testing the signal repeatability over time (precision phantoms) require high temporal and mechanical stability, along with reproducible fabrication. Phantoms that are used for training, testing, or verification purposes (accuracy phantoms) should mimic tissue properties accurately (e.g., specific tissue types, pathologies, and species of interest) either in a static or dynamic manner, to replicate expected signals. It is recommended that the bulk medium from which a phantom is prepared fulfils the following base properties^30^ (ranked in no preferential order):

• Having defined, biologically relevant properties [e.g., for PAI defined optically by the absorption and reduced scattering coefficients; and acoustically by the speed of sound and acoustic attenuation (Table 2)];
• Being well characterized using independent reference methods; calibrated against reference standards;
• Being safe to handle and to prepare in a laboratory environment with non-toxic ingredients and minimal environmental impact of materials and processes used; no expert training required for material fabrication;
• Being formed of chemical constituents that are widely available from commercial chemical vendors internationally, ideally with known intra- and inter-batch variation where available;
• Having a fabrication process possible with commonly available chemical lab equipment (Table S1 in the Supplementary Material) and basic experimental skills under protocol guidance;
• Demonstrating long-term temporal stability [>6 months (based on typical service cycles seen in commercial photoacoustic devices on the market)] of optical, acoustic, and mechanical properties (structural robustness and durability) in a realistic range of ambient room temperatures (18°C to 25°C) and humidities (30% to 80%) to allow convenient storage across the globe;
• Short-term tolerance and maintenance of structural integrity for handling and transportation in a temperature range between 4°C and 40°C based on appropriate transportation and handling procedures.

A phantom material for PAI should also maintain its structural and material integrity when in contact with an aqueous medium as water-based solutions are often used as acoustic coupling agents during signal acquisition. In addition, the material should be photostable at the visible and NIR wavelength range under safe exposure limits as encountered during imaging, handling, and storage. A suitable bulk material should further allow the embedding of target inclusions without their degradation to enable quantitative assessment of image quality metrics for specific applications. Ideally, the material should allow for the inclusion of targets made out of the same material type as well as of targets made out of different material types (e.g., microspheres, wires, etc.). Further properties should be tailored toward the system type, PA diagnostic procedure, and/or tissue type of interest. For example, for surgical training phantoms, a material type with “self-healing” properties (e.g., self-removal of applied needle tracks or cuts) may be beneficial to increase the life span of the phantom and minimize costs and manufacturing time. Phantoms for macroscopic systems may only require mimicking spatially averaged properties of biological tissue, whereas phantoms for microscopic imaging applications may need to replicate the fine structural details and heterogeneous composition of tissues to accurately support high-resolution imaging. This also necessitates adapting respective manufacturing methods (step 6).

Importantly, an ideal material should be accessible to everyone in the scientific community. If it cannot be procured in a “ready to manufacture” state at more than one standard scientific material supplier, its ingredients (including chemical abstract service numbers) and detailed manufacturing process should be openly published. Reproducible fabrication should be evidenced by a multi-center study (see step 7) to ensure that the material has broad accessibility, achieving desired properties within acceptable uncertainty limitations. An overview of tissue-mimicking materials (TMMs) proposed for PAI phantoms can be found in Table S2 in the Supplementary Material.

#### 2.4.1 Optical properties

PAI phantoms require both biologically relevant optical and acoustic properties. As optical properties vary with wavelength, a relevant base wavelength needs to be defined that enables comparison. While this is dictated by the application, 800 nm may be taken as a general option as it approximates the isosbestic point of hemoglobin. The background material should ideally be characterized by low optically attenuating values to allow tunability for a variety of tissue types.^20^ $\mu_{a}$ of $<0.02\;{\mathrm{cm}}^{-1}$ and ${\mu_{s}}^{\prime }$ of $<2\;{\mathrm{cm}}^{-1}$ may be taken as benchmark values. For specific applications, these values should be increased to match average optical values of the biological tissue of interest (Table 3). For example, for application in breast imaging, $\mu_{a}$ is recommended to be at least $0.02\;{\mathrm{cm}}^{-1}$ and ${\mu_{s}}^{\prime }$ to be at least $10\;{\mathrm{cm}}^{-1}$ (both ±10%)^20^ at 800 nm. Deviations from these recommendations may be made on an application-specific basis.

Target inclusions should exhibit a photoacoustic response at a wavelength relevant to the target system/application. Recommended wavelengths are: 532 nm (for microscopy or mesoscopic systems); 540 and 576 nm (oxyhemoglobin peaks); 758 nm (deoxyhemoglobin peak); 800 nm (isosbestic point of hemoglobin); 850 nm (above isosbestic point) and 1064 nm (many systems use fundamental wavelength of Nd:YAG lasers). The final target value is device- and application-specific, for example, PAI systems targeted toward the short-wave infrared range (>1200  nm) may require a higher value. Fluorescence effects of the material at these wavelengths are not considered, but phantoms should ideally not exhibit any fluorescence or other optical behaviors that reduce photoacoustic conversion efficiency.

Other biologically relevant properties include the anisotropy factor g, which is accounted for by ${\mu_{s}}^{\prime }$ ($=\mu_{s}(1-g)$), and the refractive index, n (see step 5 for characterization). Ideally, a PAI phantom material should exhibit forward scattering comparable to the tissue of interest, resulting in values of g>0. The refractive index n should also mimic soft tissues, if possible (Table 3).

#### 2.4.2 Acoustic properties

For acoustic properties, guidance can be taken from diagnostic ultrasound standards that exist for TMMs for various ultrasound imaging applications. For example, for conventional B-mode imaging a speed of sound of $1540\pm 15\;m\cdot s^{-1}$ and an acoustic attenuation of 0.5 to $0.7\pm 0.05\;\mathrm{dB}\cdot {\mathrm{cm}}^{-1}\;{\mathrm{MHz}}^{-1}$ (for frequency range 2 to 15 MHz,^23^^,^^31^ due to the frequency dependence of acoustic properties) is recommended. For continuous wave Doppler systems, slightly different values are advised but in overlapping ranges, with a speed of sound for blood-mimicking fluids of $1570\pm 30\;m\cdot s^{-1}$^32^ and an acoustic attenuation of 0.5 to $1.0\;\mathrm{dB}\cdot {\mathrm{cm}}^{-1}\;{\mathrm{MHz}}^{-1}$.^33^

Based on these existing values, IPASC recommends that the speed of sound of a PAI TMM should preferably lie in the range of 1430 to $1550\;m\cdot s^{-1}$,^34^ which accounts for (1) the wide range of values observed in biological tissues (Table 3) and (2) for the diversity of PAI systems requiring specific acoustic properties targeted toward the application of interest. The larger contributions of fat and water in common tissues of interest for PAI (e.g., breast tissue) legitimate an extension of the acceptable range toward the lower speed of sound.^35^ The target speed of sound should be chosen based on literature values for the application. For acoustic attenuation, the acceptable range is recommended to be 0.5 to $2\;\mathrm{dB}\cdot {\mathrm{cm}}^{-1}\;{\mathrm{MHz}}^{-1}$ based on the range of acoustic properties measured in relevant human tissues^6^ (Table 3). Deviations can be argued on an application-specific basis. The chosen values should be repeatable with high precision (see step 7).

If quantification of other acoustic or mechanical properties can be performed, such as the ultrasound non-linearity parameter (B/A), acoustic back-scattering coefficient, echo reduction, density, or Grüneisen parameter, it is recommended that the values approximate the values of the target tissue of interest (Table 3).

### 2.5 Step 5: Defining Means to Characterize the Desired Properties

Phantoms require detailed characterization of their intrinsic properties with specialist equipment that is regularly calibrated. Metrology institutes often host such facilities and several already participate in biophotonic standardization initiatives. Equipment hosted in research and industrial laboratories should ideally be cross-referenced to such reference institutes to determine the accuracy with which local characterization can be undertaken for a given material. Ideally, traceability to gold-standard metrology, such as those supported by the National Institute of Standards and Technology (NIST), should be achieved. Guidance is given here for characterizing the optical (${\mu_{s}}^{\prime }$, $\mu_{a}$) and acoustic properties ($v_{f},\alpha_{f}$) of a material. Advice on techniques to measure the density,^36^ non-linearity parameter,^37^ the acoustic back-scattering coefficient,^38^ echo reduction,^39^ and Grüneisen parameter^19^^,^^40^ can be found elsewhere as referenced.

#### 2.5.1 Characterization of optical properties

Optical properties should be verified through spectrophotometric characterization. Various methods have been proposed for optical property measurements,^41^ which can be classified according to their resolution domain: steady state domain systems (e.g., based on integrating spheres^42^^,^^43^), time domain systems,^44^ time frequency domain systems,^45^ spatial domain systems,^46^ and spatial frequency domain systems.^47^ Steady state domain systems based on integrating sphere systems are one of the most commonly used approaches for optical characterization due to their low cost, simple setup, short acquisition times, ability to characterize a broad range of optical properties, and detailed how-to-guides being readily available.^42^^,^^48^^,^^49^ Time domain optical approaches offer the highest accuracy, but they require sophisticated, costly equipment, and long acquisition times. A more detailed description of optical characterization approaches can be found elsewhere.^41^^–^^47^ Accurate characterization of optical properties in material samples has long been a significant challenge.^30^^,^^50^ Hence, the provision of raw data is crucial for enhancing confidence in the measurement results. The temperature at which the characterization measurements are performed should be reported (preferably at room temperature [18°C to 25°C]). Due to the high variability associated with optical measurements, particular care should be taken that samples are homogeneously composed with a smooth sample surface and constant thickness and that multiple (≥3) measurements per sample at different positions are performed to reduce intra-sample measurement variability. Measurements (or literature references) for the refractive index n and anisotropy factor g should ideally be given alongside the reduced scattering and optical absorption coefficients.

#### 2.5.2 Characterization of acoustic properties

Approaches for the measurement of acoustic properties (speed of sound and acoustic attenuation)^51^^–^^54^ can be broadly divided into continuous wave techniques^55^ and broadband pulse techniques.^56^ Continuous wave techniques are highly accurate and beneficial for detecting small changes in the attenuation or sound velocity but are time consuming and subject to artifacts due to reflections or other interfering signals.^57^ Pulsed techniques are often preferred for material characterization due to their easy operation, lower cost, and rapid, non-invasive measurement.^58^ They can be further categorized into the pulse-echo technique (one transducer as transmitter-receiver) and the through-transmission technique (two transducers as transmitter/receiver, respectively). Further details on measurement procedures are given elsewhere.^39^

Significant variation in results from intercomparisons^59^ have been reported historically and a number of recommendations can be identified to reduce uncertainty in acoustic characterization. It is recommended that characterization measurements are performed at room temperature (18°C to 25°C), with validated corrections being applied for measurements made outside of this range. The frequency range over which the characterization is performed must be reported. Due to the frequency-dependence of acoustic properties, Fourier transform methods are often employed in characterization analysis techniques. Acoustic characterization becomes more challenging at higher frequencies as ultrasound characterization is commonly performed in water, in which attenuation significantly increases at elevated frequencies, reducing the signal-to-noise. It is essential to limit the water path through which signals travel. Therefore, through-transmission techniques are often recommended, offering high measurement accuracy, fast acquisition speed, and ease of operation.^60^

It may be necessary to use thin plastic membranes to avoid interaction between the sample and the coupling medium,^61^ which can reflect the incident signal thus reducing the acoustic signal passing through the sample and requiring corrections to account for interfacial losses.^60^ Samples should be prepared with parallel surfaces or membranes and accurately positioned in front of the transducer to achieve planar interactions and reduce unwanted acoustic reflections and reverberations. Independent of the technique, measurements should include a robust uncertainty analysis, ideally with type A and type B uncertainty estimations.

### 2.6 Step 6: Designing a Phantom

**Fig. 3:**
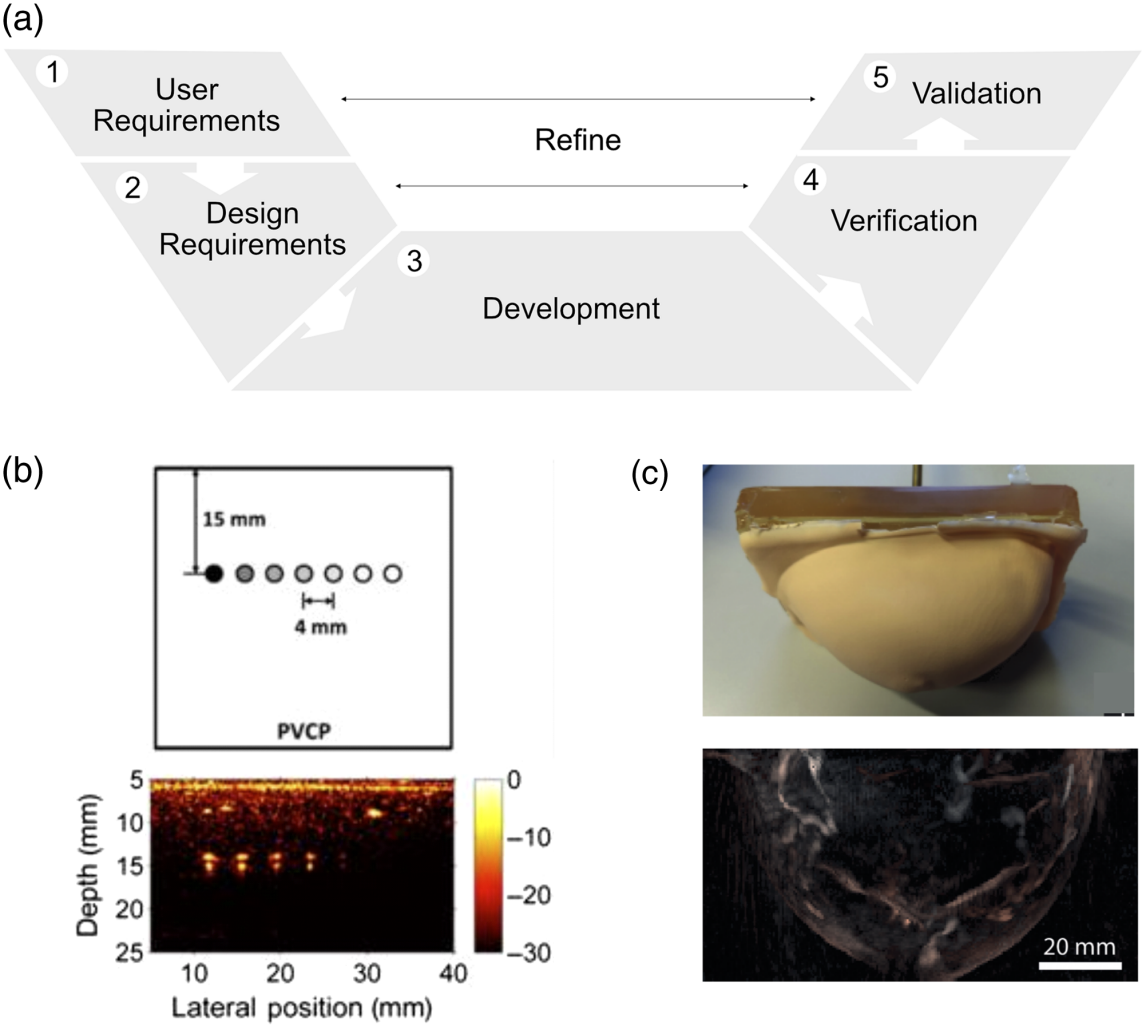
PAI phantom designs. (a) Overview of the general phantom design process. (b) Representative example of a basic image sensitivity PAI phantom: schematic (top) and PA image (bottom) of a PVCP phantom containing PTFE tubes filled with different concentrations of India ink. (c) Representative example of a more complex, anthropomorphic phantom: photograph (top) and PA image (bottom) of a breast-shaped PVCP phantom. Panel (b) adapted with permission from Ref. 62, Optica. Panel (c) adapted with permission from Ref. 63, Optica.

After defining the properties of the base phantom material, a suitable design needs to be found that allows the task of interest to be performed (Fig. 3). A phantom design is dependent on the user requirements, which—as outlined in step 3—can be manifold,^30^ ranging from user training, instrument calibration or optimization, to prototype testing. The design parameters that arise from these requirements can be: (1) qualitative (relating to the overall architecture), e.g., concerning the shape, form, size, dimensionality, or the presence of certain particular design features, such as flow circuits; or (2) quantitative (relating specific TMM properties), specifying optical $\left({\mu_{s}}^{\prime },\mu_{a},g,n\right)$, acoustic ($v_{f},\alpha_{f}$), and/or thermal (Γ) values (Table 2).

Having identified suitable design parameters, the phantom can be developed using appropriate manufacturing methods.^64^^,^^65^ These can include, but are not limited to, (1) casting and molding techniques, (2) additive manufacturing techniques (e.g., 3D-printing, layer-by-layer assembly, or lithographic techniques), and (3) subtractive and fiber formation techniques (e.g., machining and milling; electrospinning). Following phantom fabrication, the design parameters need to be verified using suitable characterization methods (see step 5). Finally, validation needs to be performed by imaging the fabricated phantom to test whether it meets its functional requirements. For simple phantoms designed for quantification of basic imaging parameters, guidance on consensus test methods best practices have been recently summarized.^66^ PAI standards for phantom designs (e.g., relating to phantom geometry, target inclusion sizes and patterns, and measurement/analysis protocols) do not yet exist but will be addressed by IPASC in future efforts to enable objective, quantitative assessment of image quality across different devices. Such standards may not only be beneficial for basic performance assessment phantoms^22^^,^^62^^,^^67^^–^^69^ but should ideally also extend to biomimetic, anthropomorphic phantoms^63^ to provide more clinically relevant image quality assessment approaches.

### 2.7 Step 7: Ensuring Reproducibility

The last—and arguably most important step—is ensuring reproducibility of the phantom manufacture. Only if a material can be manufactured reproducibly, does it have the potential to be reliably used at different centers allowing inter-device comparison and calibration. If a phantom material cannot be obtained from a commercial supplier that can certify its properties, it is recommended to conduct a multi-center study to verify the compliance of a material candidate with the aforementioned requirements. This ensures that the candidate material can be reproduced and characterized reliably using a provided protocol. For such study, three batches of the material should be repeatedly (n≥3) manufactured by ≥3 independent institutions to enable statistical analysis. It is recommended that relevant material parameters of the resulting material batches should be verified by at least two institutions to avoid bias toward a certain measurement instrument. Variations of the phantom properties should fall within specified acceptance ranges. For acoustic properties, uncertainty ranges for the speed of sound and acoustic attenuation values of TMMs have been specified before^70^ (acoustic attenuation coefficient α±7%, speed of sound v±1% within a ±1.5  MHz bandwidth). The measurement of optical properties with high accuracy and precision is more challenging, and uncertainty values will highly depend on the measurement technique used^30^^,^^42^^,^^71^ and the final properties of the phantom. For this reason, users are advised to adapt the optical uncertainty limits as appropriate to their final application.

Using ingredients available from standard scientific suppliers can help to minimize batch-to-batch variations and maximize availability. The temporal variation/stability of the material properties should be determined under prescribed characterization and material storage conditions. Properties should ideally remain stable for a time-period of at least 6 months. Accurate and precise assessment of material properties requires detailed knowledge of the characterization systems and, in particular, an assessment of the uncertainty associated with each characterization measurement.

The criteria employed for stability should be explicitly stated for each test, but it will typically involve any drift in the parameter being less than 2× the accepted uncertainty figure.

## 3 Outlook

Phantoms are crucial tools to standardize imaging systems, enabling device calibration, performance evaluation and inter-device comparisons. Particularly in newly emerging fields, such as PAI, the establishment of standardized phantoms is paramount to accelerate development and clinical translation of the technology. Here, we summarized the recommendations of IPASC on the development of phantoms in PAI, hoping to facilitate and unify methods of system testing and validation in the field. The seven-step phantom development framework presented here is targeted toward PAI but may also be applied for other imaging techniques.

While the guidance tries to be as specific as possible, systems in PAI are diverse, covering different scales and configurations and thereby impeding a “one-fit-for-all” approach. Depending on their final application, phantoms will differ in design and complexity. For early-stage technologies, such as PAI, which are not yet integrated into the standard-of-care, proposing standards for base material and testing methods, including suitable performance metrics and terminology, may be the best step forward to support the development of the technique. At later stages, device market leaders will emerge, and specific phantom types can be commercialized. Similar developments can be observed in more mature imaging technologies, such as computed tomography, X-ray mammography, ultrasound, or magnetic resonance imaging, where standards have been established through standards organizations such as the International Electrotechnical Commission (IEC), International Organization for Standardisation (ISO), and National Electrical Manufacturers Association (NEMA).^66^^,^^72^ Here, commercially available phantoms exist that are rigorously characterized by the manufacturer to ensure conformity to standards during acceptance testing, QC, and maintenance/repairs. Some of these phantoms are even traceable to gold standard metrology, such as those supported by NIST.^73^ Besides consensus in phantom development, agreement in procedures for PAI data acquisition, analysis, and metric calculation is equally important. First efforts in this direction have been already made.^66^ Along the path toward these standards, comprehensive description and documentation of data acquisition and analysis procedures is critical to ensure reproducibility.

Here, we only outline the first steps in harmonizing phantom development and testing in PAI. To move forward on the path to standardization, commitment and collaboration from all stakeholders is required.^30^ Only by aligning testing methods, translation of new technologies, such as PAI, can be accelerated, unlocking their full potential.

## Supplementary Material



## Data Availability

All data in support of the findings of this paper are available within the article or as Supplementary Material.
