# Supplementary material for: Tutorial on phantoms for photoacoustic imaging applications
Source: J Biomed Opt. 2024 Aug 14;29(8):080801. doi: 10.1117/1.JBO.29.8.080801 (PMC11324153; doi:10.1117/1.JBO.29.8.080801)
Supplement: Supplementary file 1 [file JBO_029_080801_SD001.pdf]

Supplementary Materials

Table S1 List of general laboratory equipment for phantom fabrication.

Fabrication step	Equipment examples
Mixing	Bath sonicator, vortex, (blade) mixer, mechanical stirrer
Heating	Hot plate, oven, heating bath, heating mantle
Weighing	Electronic laboratory scales / scientific balances
Handling	Glassware
Vacuuming	Vacuum oven, vacuum chamber, Vacuum Pump
Personal protection	Lab coat, googles, gloves

Table S2 Overview of materials used in PAI phantoms (adapted from Ref. 69).

Material	Advantage	Disadvantage
Polyester resin [70], epoxy resin [71]	• Optical transparency;<br>• Low solubility in water;<br>• Long term stability. [70,72]	• High sound speed and acoustic attenuation;<br>• Support fast shear waves[65].
Water/Coupling gel	• Easily obtainable;<br>• Largely optically transparent;<br>• Well defined optical and acoustic properties;<br>• Sound speed similar to biological tissue, though somewhat lower. [65]	• Cannot be used to easily sustain an insert;<br>• Does not allow tuning of acoustic properties;<br>• Speed of sound varies significantly with temperature[65] ;<br>• Allows only reduced flexibility in architecture (shape, layering).
Hydrogels e.g. agar, bovine gelatin, polyaacrylamide [55,73–75]	• Largely optically transparent;<br>• Tissue-mimicking and tuneable acoustic, optical and elastic properties;<br>• Flexibility in size, shape, and material composition;<br>• Relative ease of preparation; [75]<br>• Cost-effective;	• Absorption of water;<br>• Well-defined inserts are short-lasting due to diffusion; need for encapsulation of dyes;[76]<br>• Potential reaction with inserts (e.g., nickel and copper ions), impacting optical absorption[73];<br>• Differing dye optical absorption in solution from absorption in gelatin[74];<br>• Susceptibility to dehydration and bacterial growth in storage[65,74];<br>• High susceptibility to physical damage[65];<br>• Low temperature stability at physiological temperatures, causing structural integrity loss;<br>• Limited re-use capability, heavily conditioned by imaging, handling and storage conditions [65].
Glycerol/mineral in oil [77–83](eg. gel wax)	• Tissue-mimicking and tuneable acoustic, optical and elastic properties [78,79];<br>• Thermoreversible;<br>• Good temporal stability;<br>• Ease of use;<br>• Insoluble in water;<br>• Mechanical robustness [81].	• Speed of sound slightly lower than ultrasound standard (1440-1510 m/s vs. 1540 m/s);<br>•High temperature required for preparation (>150 °C).
Polyvinyl alcohol (PVA)[84–86]	• Tissue-mimicking acoustic, optical and elastic properties [78,79];<br>• Greater longevity and structural rigidity than hydrogels. [84,87,88]	• Extensive preparation (involves long freeze-thaw cycles);<br>• Sensitive to humidity [65];<br>• Inhomogeneities due to differential heating and cooling rates [85];<br>•Dye diffusion from inclusions has been observed after one year [87];<br>• Acoustic and optical properties are not independently tuneable [57].
Polyvinyl chloride plastisol (PVCP) [57,89–91]	• Largely optically transparent;<br>• Large Grüneisen parameter;<br>• Insoluble in water;<br>•Tissue-mimicking tuneable acoustic and optical properties[57];<br>• Stable during storage (up to 6 months) [57,89];	• Complex preparation;<br>• High preparation temperatures (180 °C), limiting usage of certain dyes and inserts;<br>• Higher acoustic attenuation than certain fat tissue types;<br>• Lack of a widely available supply chain by reference chemical suppliers.
Silicone e.g., room-temperature vulcanising (RTV), polydimethylsiloxane (PDMS) [92,93]	• Insoluble in water;<br>• Stable during storage;<br>• Variable Young's modulus, englobing biological tissue levels;<br>• Relative ease of preparation;<br>• For PDMS: Machineability and capability of creating microfluidic channels.	• High acoustic attenuation and low speed of sound (<1000 ms^{-1})[94];<br>• Organic dyes not suitable for addition[72];<br>• For RTV: high costs [72];<br>• Hardening time.

Ex vivo tissues (eg. turkey, chicken [96], or pork [97] tissue)	• Very good approximations of clinical samples;<br>• Ease of preparation;	• Not reproducible;<br>• Short-term stability;<br>• Does not allow tuning of acoustic or optical properties; limited control of phantom architecture.
